# Microarray-based uncovering reference genes for quantitative real time PCR in grapevine under abiotic stress

**DOI:** 10.1186/1756-0500-5-220

**Published:** 2012-05-07

**Authors:** João L Coito, Margarida Rocheta, Luísa Carvalho, Sara Amâncio

**Affiliations:** 1 Centro de Botânica Aplicado à Agricultura, Departamento de Recursos Naturais, Ambiente e Território, Instituto Superior de Agronomia, Universidade Técnica de Lisboa, Lisbon, Portugal

**Keywords:** Microarray, Grapevine, Real time RT-PCR, Reference gene

## Abstract

**Background:**

Quantitative real time polymerase chain reaction is becoming the primary tool for detecting mRNA and transcription data analysis as it shows to have advantages over other more commonly used techniques. Nevertheless, it also presents a few shortcomings, with the most import being the need for data normalisation, usually with a reference gene. Therefore the choice of the reference gene(s) is of great importance for correct data analysis. Microarray data, when available, can be of great assistance when choosing reference genes. Grapevine was submitted to water stress and heat stress as well as a combination of both to test the stability of the possible reference genes.

**Results:**

Using the analysis of microarray data available for grapevine, six possible reference genes were selected for RT-qPCR validation: *PADCP*, *ubiq*, *TIF*, *TIF-GTP*, *VH1-IK*, *aladin-related*. Two additional genes that are commonly used as reference genes were included: *act* and *L2*. The stability of those genes was tested in leaves of grapevine in both field plants and in greenhouse plants under water or heat stress or a combination of both. Gene stability was analyzed with the softwares GeNorm, NormFinder and the ΔCq method resulting in several combinations of reference genes suitable for data normalisation. In order to assess the best combination, the reference genes were tested in putative stress marker genes (*PCO*, *Galsynt*, *BKCoAS* and *HSP17*) also chosen from the same microarray, in water stress, heat stress and the combination of both.

**Conclusions:**

Each method selected different gene combinations (*PADCP + act*, *TIF + TIF-GTP* and *ubiq + act*). However, as none of the combinations diverged significantly from the others used to normalize the expression of the putative stress marker genes, then any combination is suitable for data normalisation under the conditions tested. Here we prove the accuracy of choosing grapevine reference genes for RT-qPCR through a microarray analysis.

## Background

Quantitative real time polymerase chain reaction (RT-qPCR) has become a mainstream research tool for the quantification of mRNA and transcription data analysis [1,2]. This method presents many advantages when compared with the more commonly used reverse transcriptase polymerase chain reaction (RT-PCR), Northern blotting and microarrays. These advantages being its higher sensitivity, specificity, broad quantification and avoidance of post-PCR processing [2-4].

Approaches such as array-based transcription profiling technologies allow the assessment of expression levels of thousands of genes in control and stress tissues. Gene redundancy is, however, a common trait of this type of analysis and requires removal. RT-qPCR technology, due to the higher sensitivity and specificity [2,4,5] is excellent to confirm non-redundant gene expression obtained through microarray analysis. However RT-qPCR itself presents several shortcomings, the most important and relevant being the need for data normalisation [1,6-9]. Normalisation and homogenization [10] are of the utmost importance in RT-qPCR as they allow the corrections of PCR reactions due to inaccurate quantification of RNA or problems related to RNA quality and purity [1,11].

RT-qPCR data normalisation is usually performed using the expression of an internal control gene [4,6,12,13]. Such a gene is also referred as a “reference gene” or sometimes a “housekeeping gene” i.e. a gene whose expression does not change under the different conditions or tissues under investigation [1,14,15]. Unfortunately no such gene exists, and the universality of such an ideal gene is not valid, since the transcript levels of all genes show some degree of variability under different experimental conditions [5-7,16]. Initially, gene stability was assessed with the ΔCq method. In this method, the Cq value of the gene of interest (either target or reference) is related to a control/calibrator [17]. In order to overcome these flaws, statistical algorithms such as GeNorm [6] and NormFinder [18] have been developed to evaluate the best suited reference gene or a combination of genes for normalisation of RT-qPCR data in a specific set of biological data [6].

The identification of suitable reference genes can be difficult. Several attempts have been made, all with different outcomes. Studies usually allocate different “model” genes to be used in data normalisation. Usually the allocated genes vary with the plant species, as well with the experimental conditions. Also the method to select the reference candidate varies with the availability of data for the plant species under study. Studies regarding reference genes often employ a variety of methods to chose possible reference genes; such as searching the bibliography for published references [19], using orthologs of Arabidopsis reference genes [20], cDNA libraries [21] or analysis of EST libraries [22]. Genes that are frequently identified to be good references include elongation factors 1-α (*eEF-1α*) [23-26], actin (*act*) [8,15,21,23], ubiquitin (*ubiq*) [8,25,27-29], glyceraldehyde 3-phosphate dehydrogenase (*GAPDH*) [7,25,27,28], ribosomal proteins [7,21,25,30-32], SAND family protein (*SAND*) [20,27,33] and, other less common genes have been identified to be particularly good references in very specific contexts.

Czechowski et al. [5], using data from Affymetrix ATH1 whole-genome GeneChip, proposed not only the typical reference genes for RT-qPCR but also new ones. After that study, microarrays, when available, have been used for identifying reference genes [34,35], with new genes being found for RT-qPCR data normalisation.

In grapevine, several attempts have been made in identifying reference genes. Gamm et al. [36] indicated two genes (V-type proton ATPase 16 kDa proteolipid subunit and 60S ribosomal protein L18) as being optimal reference genes for the study of the expression of genes involved in pterostilbene synthesis in grapevine leaves infected by *P. viticola* and berries infected by *B. cinerea*. Reid et al. [23] also tested possible reference genes specifically suitable for use in grapevine berry development studies and suggested *GAPDH*, *act*, *eEF-1α* and *SAND* as the most stable.

Whatever the method used for choosing possible reference genes for data normalisation, stability analysis should always be performed in the optimal conditions.

Grapevine (*Vitis vinifera* L.) is a sessile organism and therefore cannot avoid abiotic stress. Plants have been developing mechanisms to cope with environmental changes and help to overcome them. The most relevant abiotic stresses that can affect the production of a Mediterranean crop such as grapevine are: drought, excessive light and excessive heat. In fact, climate models predict an intensification of extreme conditions, which can reduce production to below the threshold for optimal grapevine growth [37]. This represents a serious challenge for Mediterranean agriculture.

The pattern of gene expression in response to abiotic stress has been monitored in *ex vitro* grapevine plants by comparing the use of the grapevine Affymetrix GeneChip with extensive RT-qPCR analysis [38]; and in greenhouse and field plants through genechip microarray (results under analysis).

In this paper we report several putative reference genes chosen from a grapevine microarray analysis and identify the genes to be used as references for RT-qPCR normalisation, after obtaining the “optimal combination” of reference genes using three different methods. We also apply the three “optimal combinations” obtained to quantify the expression of a set of stress-marker genes.

## Results and discussion

### Choice of reference genes

Candidate reference genes were chosen from a microarray analysis using an array composed of 23 096 unigene sequences [39]. All the genes that did not meet the selection criteria (100% presence in control and in stress samples and both probesets present in all the stress samples of the array) were discarded. Fold-change was then analysed and all probesets outside the fold-change interval of −1.25 to 1.25 were also discarded. This range was the minimum interval in which an acceptable number (eighteen) of possible reference genes could be found (Table 1).

**Table 1:** Possible reference genes retrieved from the microarray analysis

**Probeset ID**	**NCBI Reference**	**WS fc**	**HS fc**	**Annotation**
VVTU3078_at	XM_002274960	1.04	1.02	growth-on protein GRO10
VVTU5951_at	XM_002276120	1.13	1.12	F-box protein 7
VVTU21677_at	XM_002278540	1	1.23	ATSLY1
VVTU38193_s_at	XM_002284329	1.16	1.19	RAB GTPase ARA3
VVTU775_at	XM_002283960	1.21	1.16	Aladin
VVTU38174_at	XM_002274483	−1.11	−1.04	DnaJ homolog, subfamily B, member 4
VVTU39962_s_at	XM_002265755	−1.02	−1.12	ubiquitin-like domain containing CTD phosphatase 1
VVTU291_at	XM_002273137	−1.06	−1.11	peptidylprolyl isomerase PAS1 (PASTICCINO 1)
VVTU15254_at	XM_002275607	−1.05	−1.07	DNA polymerase eta subunit
VVTU15763_at	XM_002282403	−1.02	−1.03	translation initiation factor eIF-3 subunit 4
VVTU16514_at	XM_002278163	−1.11	−1.04	Protein kinase PKN/PRK1
VVTU3178_at	XM_002271296	−1.05	−1.05	translation initiation factor eIF-2B alpha subunit
VVTU12062_at	XM_002274698	−1.01	−1	RNA-binding protein Musashi
VVTU6197_at	XM_002269673	−1.13	−1.06	plectin (myosin-like)
VVTU2620_at	XM_002282316	−1.08	−1.03	DNA repair protein RAD23
VVTU3027_at	XM_002266331	−1.09	−1.21	ankyrin repeat family protein
VVTU5961_s_at	XM_002284235	−1.22	−1.09	ribosomal protein L27
VVTU1226_at	XM_002277764	−1.03	−1	Plastid-specific 50S ribosomal protein 6

Probeset ID, NCBI Reference, Water Stress fold change (WS fc), Heat Stress fold change (HS fc) and annotation of the 18 genes retrieved from the microarray analysis [39]. The presence call is 100% in all genes in both treatments.

From those eighteen genes, six were selected for the study (Table 2). The selection was performed by choosing genes previously described or belonging to gene families commonly used for RT-qPCR data normalisation, such as Translation initiation factors; but also included grapevine genes that had not been completely described or with unknown functional categories. Well known and described reference genes were also added to the study for comparison, *act* and *L2* (Table 2).

**Table 2:** **Primers used for the reference genes chosen after analysis of Table **1

**Probeset ID**	**Name**	**Sense primer**	**Anti-Sense primer**	**pb**
VVTU3078_at	PADCP	5' ATTCATTAAAGTACCTTTCTTT 3'	5' AACACCCAAAAGATGTCGTA 3'	240
VVTU39962_s_at	*ubiq*	5' CAATTTCCTGAGTTCTACAGTT 3'	5' CCTCATTGTATGACTCCCAGT 3'	229
VVTU15763_at	*TIF*	5' AAAGCAGAAGAAACCAAGATT 3'	5' TTGCCAGTGCCTGTAGTAGCC 3'	206
VVTU3178_at	*TIF-GTP*	5' AGCAGCACAGAATAAGAAACT 3'	5' CCATCAGCCCCAACAAATACC 3'	177
VVTU3027_at	*VH1-IK*	5' CAGGGATTATGATAGTAGGA 3'	5' TTGTTTGGTAGAGGAGGTGG 3'	252
VVTU775_at	*aladin-related*	5' CCTACACTTATTCATCTTCG 3'	5' ACTTGTGGCGGTTGCTCTGC 3'	224
-	*act*	5´ TGGATTCTGATGGTGTGAGTC 3'	5' CAATTTCCCGTTCAGCAGTAGTGG 3'	167
-	*L2*	5' TCTACTTCAACCGATATGC 3'	5' CAACCTGTCCGACTG 3'	196

Probeset ID (of all but *act* and *L2* that were not chosen from the microarray), gene name, sense primer, anti-sense primer and transcript length of the product obtained.

#### Selection of the best reference genes

The first approach used to verify the stability of the eight reference genes was the ΔCq method [17] (Table 3). In this method, the control/calibrator can be any sample, *e.g*. a real untreated control, or the sample with the highest level of expression (lowest Cq value). The method generates raw (non-normalised) expression values, which need to be normalised by dividing with a proper normalisation factor. The ΔCq method has several advantages, namely, it allows an easy inclusion of multiple reference genes for normalisation. In this research, the best reference genes for RT-qPCR data normalisation obtained with this method were *PADCP*, with *act* ranked second, whilst *aladin-related* was the worst choice.

**Table 3:** Ranking of reference genes using ΔCq and GeNorm

	**ΔCq**		**GE Norm**		
			**M values of Ref genes**		
**Ref. Genes**	**Sum of Ref Gene values**	**Ranking**	**Greenhouse**	**Field**	**All samples**
***act***	**40**	**2**	**1.834**	**0.503**	1.88
***L2***	137	5	**3.604**	**0.735**	**3.55**
***VH1-IK***	408	7	1.974	**0.496**	1.884
***aladin-related***	**1243**	**8**	2.166	0.603	2.072
***TIF***	200	6	1.921	0.552	**1.829**
***TIF-GTP***	110	4	1.89	0.594	1.837
***PADCP***	**39**	**1**	2.092	0.674	2.007
***ubiq***	54	3	**1.885**	0.58	**1.817**

Ranking of reference genes using two different methods, ΔCq and the software GeNorm (http://medgen.ugent.be/~jvdesomp/genorm/). The normalisation with ΔCq was performed with all samples while with GeNorm it was performed with all samples, with greenhouse samples and with field samples individually.

The GeNorm application for Microsoft Excel determines the most stable reference genes from a group of genes. The application also calculates a normalisation factor of gene expression for each sample, based on the geometric mean of a user-defined number of reference genes. It is based on the assumption that the expression of two ideal reference genes will always have the same ratio among samples regardless of the experimental conditions [6]. This average expression stability value (M) is calculated using the expression data for each gene. M is the average pairwise variation (V) of one gene compared with each of the other reference genes tested. Stepwise exclusion of the gene with the highest M value allows the ranking of the tested genes according to their expression stability, until the two most stable genes in the remaining set cannot be ranked any further. GeNorm also allows estimating the optimal number of reference genes which should be used for normalisation. It calculates the normalisation factor (NF) based on the geometric mean of the expression of more than one reference gene. GeNorm calculates the NF_n_ of the two most stable reference genes based on the geometric mean of the expression data, and then the NF_n+1_ with the next most stable gene. To determine how many genes should be used for accurate normalisation, the pair-wise variation (V_n/n+1_) is calculated for each two sequential normalisation factors (NF_n_) and NF_n+1_[6].

**Figure 1:**
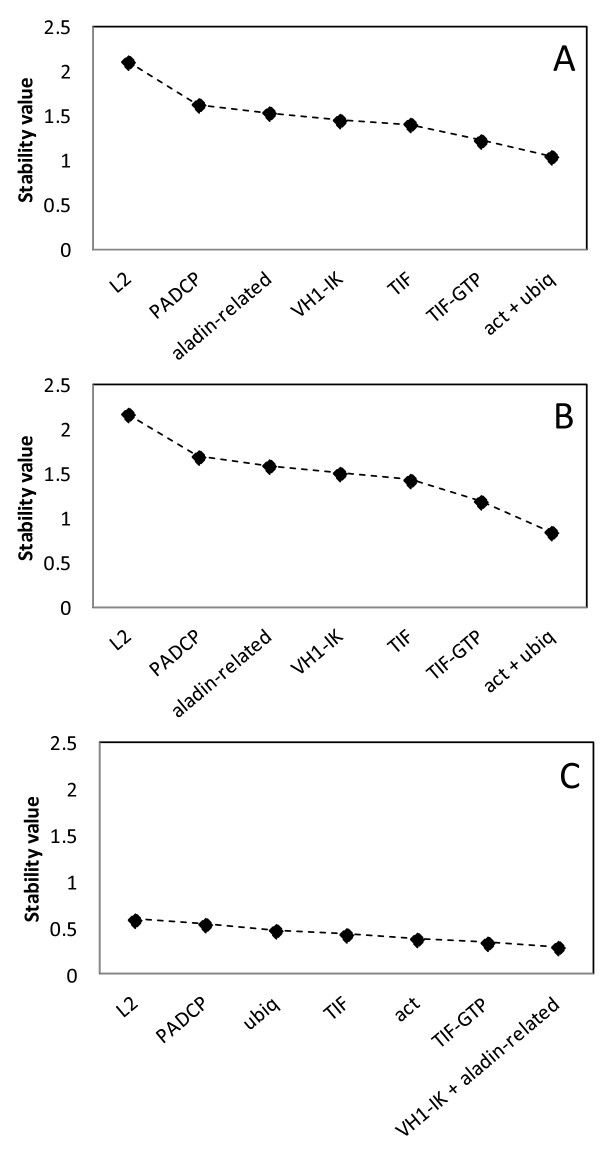
**Stability values of the putative reference genes using the software GeNorm.** Stability values of reference genes calculated using the software GeNorm, A: all samples; B: greenhouse samples; C: field samples. Because GeNorm does not analyse samples in groups, these were obtained manually.

As GeNorm does not allow the analysis of results within groups, our samples were divided manually in three groups: “all samples”, “greenhouse” and “field” (Table 3). The stability values of the eight reference genes were calculated for those three groups (Figure 1). With all samples considered, the two best possible reference genes were *ubiq*, ranked first and *TIF*, ranked second (Table 3). However, when the stepwise exclusion of the genes with the highest M value was performed, *act* and *ubiq* were considered the best option for normalisation, with a stability value around 1.00 (Figure 1A). When the greenhouse plants were considered alone, *act* ranked first and *ubiq* ranked second (Table 3). Also, after the stepwise exclusion of the genes with the highest M value, the remaining best two genes were *act* and *ubiq* (Figure 1B). When only the field plants were considered, *act* ranked first and *VH1-IK* was second, and M values were lower than those of the previous analyses (Table 3). In this case, the best group of genes included *VH1-IK* and *aladin-related* (Figure 1C). In all these comparisons, *L2* ranked as the least stable reference gene (Table 3).

While GeNorm stepwise finds the two genes whose expression ratio shows the least variation in relation to the other genes; NormFinder finds the single gene with the most stable expression and the best pair of genes with the most stable combined expression [18]. The NormFinder pair can compensate in a way that, for best performance, a gene that is slightly overexpressed in a treatment group, and slightly underexpressed in the untreated group, can be combined with a gene with the opposite bias. Furthermore, NormFinder can account for the heterogeneity in the tested samples, allowing the comparison of different treatment groups. It can thus distinguish between stability and bias [18] eventually being able to discard a candidate reference gene that is excellent for all treatment groups but one. This indicates that the particular treatment affects that reference gene, which is then not such a good candidate. These are the most striking differences between the two analysis softwares, and that they can account for different results when comparing them.

**Figure 2:**
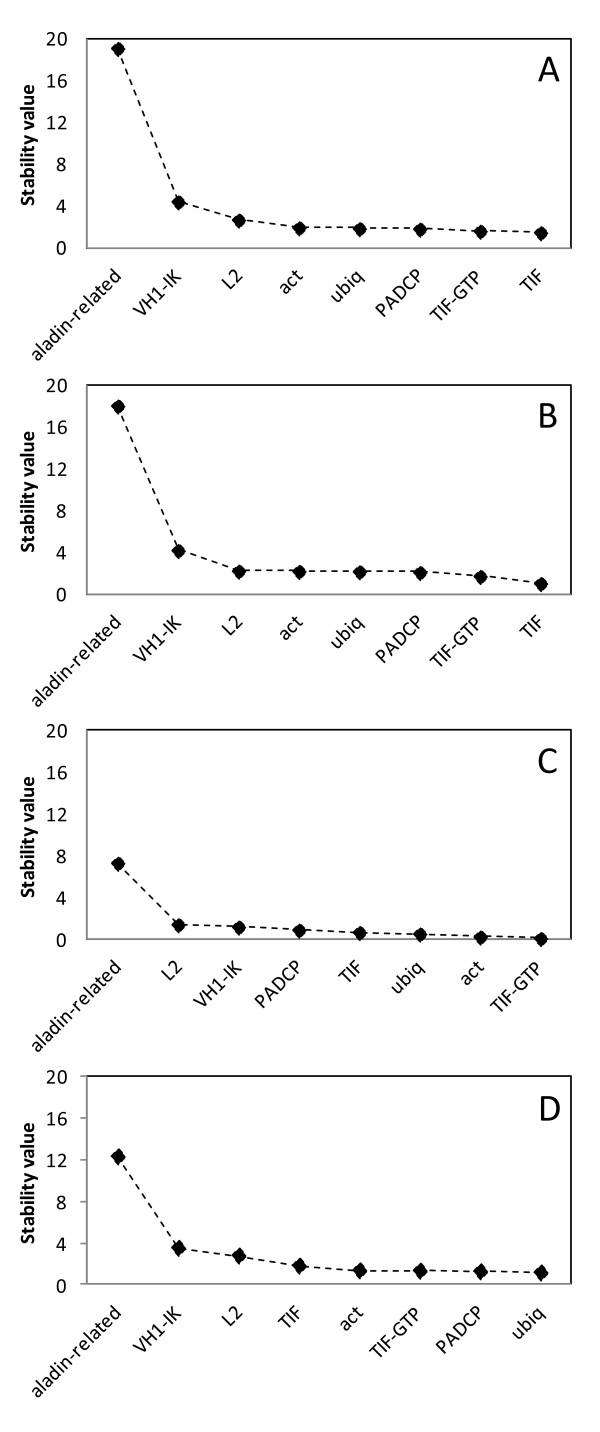
**Stability values of the putative reference genes using the software NormFinder.** Stability values of reference genes calculated using the software NormFinder, A: all samples; B: greenhouse samples; C: field samples; D: greenhouse versus field samples.

NormFinder analysis when performed without groups ranked *TIF* as the best gene for data normalisation (Table 4) as shown by the stability values (Figure 2A). The second most stable gene in this analysis was *TIF-GTP*. When only the greenhouse plants were analysed, the best and the second best ranked genes were the same (Figure 2B). By analysing field plants, the best gene was *TIF-GTP* while *act* ranked second (Figure 2C). When “Greenhouse “and “Field” groups were introduced, the most stable gene was *ubiq,* while *PACDP* ranked second (Figure 2D). However NormFinder also calculates the best combination within a group and indicated *TIF-GTP* and *TIF* as the best combination (Table 4).

**Table 4:** Ranking of reference genes using NormFinder

	**All samples**	**Greenhouse**	**Field**	**Greenhouse vs field**
**Best gene**	*TIF*	*TIF*	*TIF-GTP*	*ubiq*
**Best Combination**	*-*	*-*	*-*	*TIF + TIF-GTP*

Results of the reference genes ranking using the software NormFinder (http://www.mdl.dk/publicationsnormfinder.htm). The normalisation was performed with all samples, separating greenhouse and field and using Greenhouse and Field as “groups”. When no “groups” are introduced the result is only one best gene.

In all the GeNorm analyses *L2* was the worst performing reference gene (Table 3 and Figure 1) while *aladin-related* was the worst performing reference gene in the ΔCq and NormFinder analyses (Table 3 and Figure 2).

#### Optimal number of genes for RT-qPCR data normalisation

**Figure 3:**
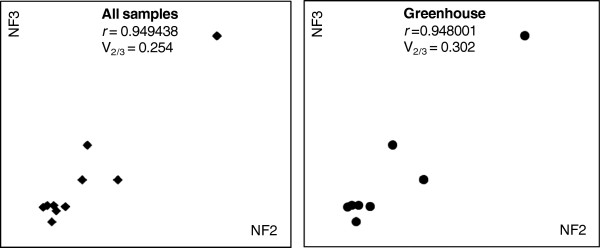
**Optimal number of reference genes required for effective normalisation.** Optimal number of reference genes required for effective normalisation. The pairwise variation (V_n_/V_n/n+1_) was analysed between the normalisation factors NFn and NFn + 1 using the software GeNorm to determine the optimal number of reference genes required for RT-qPCR data normalisation in three different situations (n and n + 1 as in the ranking of Table 3).

There is an adequate number of reference genes to be used in each experiment, which depends on the balance between stability, accuracy and some practical aspects such as time and costs [6]; because when the majority of genes is stable, the addition of further genes would be a waste of resources. It is therefore essential to find the optimal number of reference genes. It has been suggested that three is the minimal number required for a correct normalisation [6]. Further addition of reference genes should be halted when the normalisation with NF_n_ and NF_n+1_ have similar values [6]. GeNorm performs this analysis automatically by calculating the V_n/n+1_ values between each combination of sequential NF. A cutoff of 0.15 is recommended, the inclusion of an additional reference gene below this value does not result in a significant improvement of the normalisation. Yet this is not an absolute value and can change according to the data [6]. In our experiment, the pairwise variation values for all samples and greenhouse samples were above the recommended cutoff value. When the analysis was performed using the field plants, there was no increase or decrease of relevance in adding more than two genes, possibly indicating that these plants have a very stable gene expression (Figure 3). This result, although unexpected, is in agreement with the results obtained with microarrays performed in grapevine field plants at summer with high light exposure, high temperature and water shortage conditions which showed higher gene stability than greenhouse plants that were individually subjected to the same types of stresses (Rocheta et al, in preparation). One explanation for this outcome is the better adaptation capacity of fully grown and well rooted plants.

**Figure 4:**
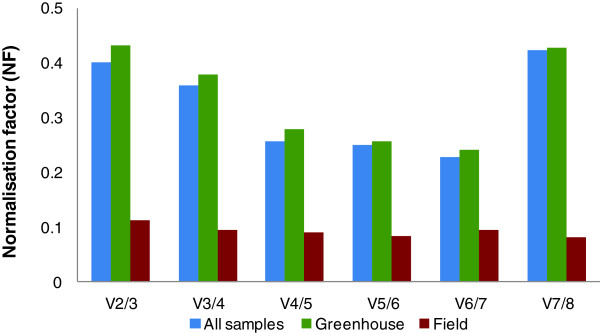
**Selected scatterplots of normalisation factors before (*x*-axis) and after (*y*-axis) inclusion of an (*n* + 1)th control gene in all samples and in greenhouse samples.** Low variation values (*V*) correspond to high correlation coefficients (*r* = Spearman rank correlation coefficient). It is clear that there is no need to include more than three control genes for both “all samples” and “greenhouse” samples.

The analysis performed by GeNorm were established based on a Spearman correlation between NF_n_ and NF_n+1_, in which low variation values correspond to high correlation coefficients. As the pairwise variation values for “all samples” and “greenhouse” samples were above the recommended cutoff value (Figure 3) we performed Spearman correlation tests ourselves and obtained the results shown on Figure 4. These results show that the addition of a third reference gene to perform normalisation does not provide relevant information, so the use of two genes is enough to accurately normalise the expression of genes of interest in those conditions.

#### Specific markers of abiotic stress

For the normalisation tests, we chose as best combinations of reference genes (BC) the following: GeNorm: *act + ubiq*; NormFinder: *TIF + TIF-GTP;* ΔCq: *PADCP + act*. *L2* was also used for comparison as the most unstable gene.

For the more up and down-regulated genes, we used probesets with the highest or lowest fold change in each stress condition. That is to say, the most up or down-regulated expressed probesets in drought could not be within the significantly expressed probesets in heat stress and vice-versa. From the two probesets chosen for drought (WS) and heat stress (HS) one was up-regulated and the other down-regulated (Table 5). The expression of these genes was tested in the leaves of plants under WS, HS or a combination of both stresses (WSHS). Primers for those genes are shown in Table 6.

**Table 5:** Putative stress marker genes retrieved from the microarray analysis

**Name**	**Probeset ID**	**Accession**	**Regulation**	**WS fc**	**HS fc**
*PCO*	VVTU27646_s_at	XM_002284733	Down	−12.14	−1.44
*GalSynt*	VVTU3450_at	XM_002262615	Up	49.98	22.25
*BKCoAS*	VVTU16209_at	XM_002284475	Down	−3.44	−14.53
*HSP17*	VVTU13941_at	XM_002267919	Up	2.92	292.07

Gene name, probeset identification (ID), NCBI reference, fold change of the most differentially expressed genes (*i.e.,* highest and lowest fold change values) in (WS) and heat (HS) obtained from the analysis of the microarray [39].

**Table 6:** Primers for the putative stress marker genes

**Name**	**Sense primer**	**Anti-Sense primer**	**pb**
*PCO*	5' GCGTCTCATTATCGTTGGTTC 3'	5' CAGTGTCCTCGTGGTATCG 3'	233
*GalSynt*	5' CCAATCCCTTCTGAATACAACC 3'	5' TTTCCCACCATTTCCTCACC 3'	184
*BKCoAS*	5' TGCGACAAGGGCTTTCATC 3'	5' CAGGCTCCAGATCATACTCAG 3'	245
*HSP17*	5' AGAAGAAGAGCCAGAAGAGAAG 3'	5' ACACACGAAGCGACCAAG 3'	250

Gene name, sense primer, anti-sense primer and transcript length of the putative stress marker genes retrieved from the microarray analysis [39].

**Figure 5:**
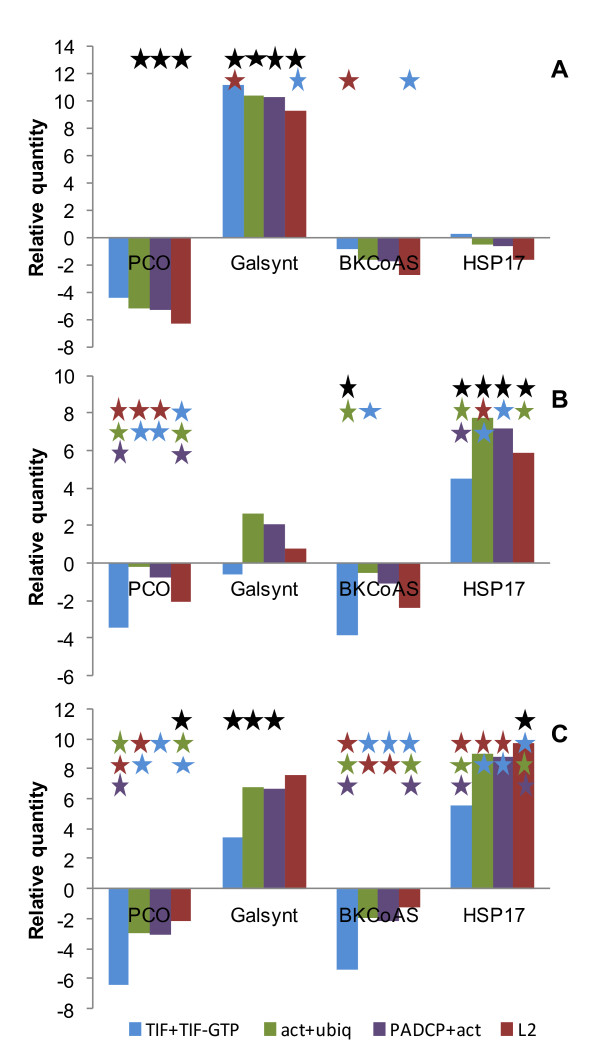
**Expression of the stress marker genes in grapevine normalised with the different Best Combinations or *L2. ***Expression of the four possible abiotic stress marker genes in grapevine plants subjected to WS (A), HS (B), a combination of both (C): *PCO* – negative marker of WS; *Galsynt* – positive marker of WS; *BkcoAS* – negative marker of HS; *HSP17*– positive marker of HS. Expression was normalised using the three Best Combinations of genes and the worst possible gene (*L2*). Significant differences (p < 0.05) are represented by ★ when compared to the opposite treatment in the case of WS and HS and in comparison with the respective single treatment in the case of WSHS. ★ of different colours represent the significant differences of the gene expression with the correspondent colour of the reference genes combination.

*PCO* (protochlorophyllide oxidoreductase) catalyses one of the steps of chlorophyll biosynthesis [40]. In the microarray analysis that supports this experiment, *PCO* was the most down-regulated gene under WS and therefore, it was chosen as a down-regulation marker of WS. In this experiment, *PCO* was down-regulated in both WS and HS (Figure 5A and B, respectively), but statistically significant differences of *PCO* expression in both treatments were only found when *PCO* was normalised with the combinations *act + ubiq*, *PADCP + act* and *L2*. In this case the use of those two BCs would be preferred to the *TIF + TIF-GTP* combination, which was unable to detect differences in *PCO* expression between both treatments (Figure 5A and B respectively).

*GalSynt* (Galactinol synthase) catalyses the first committed step in the biosynthesis of the rafinose oligosaccharide family (RFOs) and plays a key regulatory role in carbon partitioning between sucrose and RFOs [41]. In Arabidopsis the *GalSynt* isoform AtGalSynt 1 is induced by drought and salinity [42]. In the microarray analysis this experiment was based upon, *GalSynt* was the most up-regulated gene under WS, and was therefore chosen as an up-regulation marker of WS. Indeed *GalSynt* showed significant up-regulation with all the BCs and *L2* under WS (Figure 5A); while in HS it was down-regulated when normalised with the *TIF + TIF-GTP* combination and showed a slight up-regulation when normalised with the remaining BCs and *L2* (Figure 5B). The results obtained in HS were significantly lower than the expression levels verified in WS with all the BCs and *L2*. This suggested that not only this gene responds well to the treatment imposed, but that all the BCs are stress insensitive and therefore suitable for data normalisation.

*BKCoAS* (β-ketoacyl-CoA synthase) is one of the enzymes responsible for the elongation of fatty acid chains. Very-long-chain fatty acids (VLCFAs) are fatty acids that have 20 or more carbon atoms. In plants, VLCFAs are the precursors of several lipids, such as cuticular waxes, and their derivatives act as protection barriers, they also provide energy storage in the seeds and act as signalling molecules, responding to several stress stimuli [43]. In this work, the down-regulation of *BKCoAS* was studied as a possible marker of HS. The expression of *BKCoAS* under HS was down-regulated when normalised with all BCs and *L2*, as expected (Figure 5B). However under WS *BKCoAS* also displayed down-regulation when normalised with all BCs and *L2* (Figure 5A). Statistical analysis showed significant differences in the expression of *BKCoAS* between WS and HS (with the expression of *BKCoAS* being lower in HS) only when the gene expression was normalised with the *TIF + TIF-GTP* combination; making this combination ideal as a reference for RT-qPCR data normalisation in this case.

The accumulation of heat shock proteins (HSPs), under the control of heat stress transcription factors (HSFs), is known to play a central role in the response to heat stress and in acquired thermotolerance in plants and other organisms [44,[45]. We have used the gene *HSP17* as an up-regulation marker of HS. Under HS *HSP17* was up-regulated with all the BCs and *L2* (Figure 5B). Under WS *HSP17* showed differential expression, being up-regulated when normalised with the *TIF + TIF-GTP* combination and down-regulated with the remaining BCs and *L2* (Figure 5A). Statistical analysis showed that the expression of *HSP17* was significantly higher in HS than in WS, when normalised with all BCs and *L2*, therefore making any BC and *L2* suitable for RT-qPCR data normalisation.

Gene expression of the chosen markers was also tested under combined WS and HS conditions (Figure 5C) in order to evaluate the reliability of the genes chosen as stress markers in a complex environment when more than one factor is at stake. All the genes used as stress markers responded as expected, with *PCO* down-regulated in response to WS and *BKCoAS* was down-regulated due to HS. Similarly, *GalSynt* was significantly up-regulated in WS, whilst *HSP17* was up-regulated in HS. When statistical analysis was performed between the expression values of the stress marker genes under WSHS and their expected individual stress response; we found that *PCO* expression, when normalised with the combinations *act + ubiq*, *PADCP + act* and *L2,* was significantly lower when compared with its expression under WS. In this case, the best combination for data normalisation would be *TIF + TIF-GTP*. Under WSHS, *GalSynt* expression showed significant differences in relation to WS when normalised with the three BCs. In this occasion, *L2* would be the most suitable gene for data normalisation, probably due to the expression stability of *GalSynt*. Under WSHS no significant differences were found in *BKCoAS* when compared to the expression of this gene in HS; rendering all BCs suitable for data normalisation. Under WSHS *HSP17* showed a significantly higher level of expression when compared to HS, only when *L2* was used as reference, making it a desirable reference gene in such situation.

Most works propose different combinations of reference genes for different experimental conditions after combining the best reference genes that present a ranking consensus between different methods [15,25,27]. Conversely, our reference genes were selected to be used in analysing gene expression after different treatments. We used the BCs obtained and tested them individually by method. In fact, if we had used the four best ranking genes of all treatments and the three methods, we would have obtained the same four reference genes that comprise the three BCs. Therefore we propose that the BCs obtained with any of the methods are suitable reference genes for transcription studies and this option is obviously less time and resource consuming than the use of all four genes.

Regarding the most stable genes, *ubiq* is referred as a reference gene in scientific works [8,21,25,27], as confirmed in the microarray analysis, although its presence was recorded in only one combination. *act* was chosen due to its common use as reference gene [8,21,23] and was present in two of the three combinations. Translation initiation factors have been tested by other authors but without success [17,19], while in our study *TIF* and *TIF-GTP* showed good stability and their combination was selected by NormFinder. *PADCP* is, to our knowledge, referred to for the first time as a potential reference gene and not only showed stable expression in the microarray analysis, but was also selected as reference gene by the GeNorm software. Ribosomal protein genes have been used as references in several studies [7,19,31] while in others they were ruled out as not stable enough [24,29]. *L2*, as one of those genes, was considered a good reference in one study [46] but did not perform so well in another [26]. In our work *L2* ranked as the least stable gene; however in some situations when normalizing stress markers, it outperformed the BCs, highlighting its major problem of being inconsistent.

## Conclusions

This study attempts to provide the validation of reference genes in grapevine leaves under abiotic stress conditions for RT-qPCR data normalisation. The availability of a custom microarray for grapevine was of great assistance in the choice of candidates. We used plants submitted to water and heat stresses, as well as a combination of both. Six genes that presented high levels of stability in the microarray were tested in RT-qPCR, as well as two other genes commonly used in RT-qPCR data normalisation. Through the use of GeNorm, NormFinder and ΔCq methods, we obtained three possible reference gene combinations: *act + ubiq*; *TIF + TIF-GTP* and *PADCP + act*. With regards in obtaining the best combination of genes resulting from the analysis with the different softwares and methods, it was not possible to appoint a single optimal combination lining off from the others. All combinations of reference genes were able to normalise the putative stress markers; but not without flaw. Therefore we can conclude that any of the combinations tested is suitable to be used as reference for RT-qPCR data normalisation of grapevine leaf samples under abiotic stress; we prove that microarray analysis can be a powerful tool to obtain reference genes. When microarrays are not available some of the most commonly used references (*act* and *ubiq)* are in fact good options, but this choice must be taken with caution because some are not (*e.g. L2*). The careful testing of reference genes also comes out as paramount when compared to the test method. In fact, the three alternatives evaluated here performed equally well.

## Methods

### Greenhouse plant material and stress treatments

Cuttings from pruned wood of pre-selected plants of the variety Trincadeira were grown in pots in the greenhouse under the following controlled conditions: 200 μmol quanta m^-2^ s^-1^ irradiance, 16 h light/8 h dark photoperiod, temperature of 25°C day/ 23°C night and well watered with nutrient solution whenever necessary.

Individual stresses were applied when shoots were 50 to 60 cm high. The stresses applied were: HS – 1 hour at 42°C; WS – stop irrigation until the pre dawn leaf water potential (Ψ_w_) was −0.9 MPa and WSHS – a combination of both. Ψ_w_ was measured with a pressure chamber, Model 600, PMS Instruments Company (Albany, OR). Samples consisted of the third, fourth and fifth totally expanded leaves and were taken immediately after the end of the stress (or, in the case of WS, after the measurement of the pre dawn leaf Ψ_w_), frozen in liquid nitrogen and stored at −80°C[44] until RNA extraction.

### Total RNA extraction and cDNA synthesis

Samples were ground with a mortar and pestle in the presence of liquid nitrogen. Total RNA was extracted with the RNA Plant Total RNA Kit (Sigma-Aldrich, Inc) following the manufacturer’s instructions. Nucleic acid concentration of each sample was quantified by spectrophotometry using the software Gen5 1.09 (Synergy HT, Bio-Tek Instruments, Winooski, USA). Total RNA quality was assessed using the A_260_/A_280_ and A_260_/A_230_. Only RNA samples with A_260_/A_280_ between 1.8 and 2.1 and A_260_/A_230_ between 2.0 and 2.2 were used. Total RNA integrity was checked through 1% agarose gel electrophoresis under denaturing conditions.

RNA samples were treated with RQ1 RNase-Free DNase (Promega, Madison, WI). cDNA was synthesized from 2 μg of total RNA using oligo(dT)_20_ in a 20 μL-reaction volume using RevertAid Reverse Transcriptase (Fermentas Life Science, Helsingborg, Sweden) according to the manufacturer’s recommendations. cDNA was tested for gDNA contamination in PCRs using the intron spanning primers *ubiq* (Table 2) that yield a 229 bp amplicon in cDNA and a 547 amplicon in gDNA. Amplicon sizes were compared in 2% agarose gels together with the molecular weight marker 1Kb + (Invitrogen) and no gDNA contamination was detected. cDNA was stored at −20°C until further use.

### Selection of reference genes and primer design

The selection of the possible reference genes for RT-qPCR was made based on a previous microarray analysis performed with two biological replicates for each experimental condition (data not shown) using a 23 096 unigene sequences array [39] based on the lower fold-change, function, category and presence in all replicates. The fold-change chosen was within the interval of −1.25 to 1.25, which is the smallest interval where sufficient possible reference genes could still be detected. The possible reference genes were selected from this pool, taking into account their category and function in order to obtain a mix of genes. This included members of families commonly used as reference genes in RT-qPCR data normalisation and other putative genes which do not have a clearly described function or which function remains unknown in grapevine but nevertheless displayed high stability on the microarray analysis. Six possible reference genes were selected from this group and two typically used reference genes were added to the study for comparison, *act* (AF369525.1) and *L2* (AJ441290.2).

Primers for these eight putative reference genes were designed using the software Primer Premier 5.0 (Premier Biosoft International) using a primer length of 20 ± 2 bp, melting temperature of 60°C ± 2°C, a guanine-cytosine content of *circa* 50% and an expected amplicon size of 180–280 bp.

### Real-Time PCR

The real-time PCR was performed in 96 well white reaction plates (Bio-Rad, Hercules, CA), using an IQ5 Real Time PCR (Bio-Rad, Hercules, CA) with three biological replicates and two technical replicates. The 20 μL reaction mixture was composed of 1 μL cDNA diluted 50-fold, 0.5 μM of each gene-specific primer and 10 μL master mix (SsoFast_EvaGreen Supermix, Bio-Rad, Hercules, CA). Amplification of PCR products was monitored via intercalation of Eva-Green (included in the master mix). The following program was applied: initial polymerase activation, 95°C, 3 min; then 40 cycles at 94°C 10 s (denaturation), 60°C 20 s (annealing), 72°C 15 s (extension). The PCR products were run on 2% agarose gels to make sure that there was only one amplicon of the expected size. PCRs with each primer pair were also performed on samples lacking cDNA template, in triplicate (no template controls).

To assess amplification efficiency of the candidate genes, identical volumes of cDNA samples were diluted and used to generate five-point standard curves based on a five-fold dilution series (1;1:5;1:25;1:125;1:625), in triplicate. Amplification efficiency (E) is calculated as E = 10^(−1/a)^-1, “a” being the slope of the linear regression curve (y = a log(x) + b) fitted over the log-transformed data of the input cDNA dilution (y) plotted against the respective quantification cycle (Cq) values (x). E-values of the target genes were considered comparable when they did not exceed 100 ± 10%, corresponding to a standard curve slope of 3.3 ± 0.33. All cDNA samples were diluted 50 fold and were amplified in duplicate in two independent PCR runs.

To generate a baseline-subtracted plot of the logarithmic increase in fluorescence signal (ΔRn) versus cycle number, baseline data were collected between the cycles 5 and 17. All amplification plots were analysed with an *R*_*n*_ threshold of 0.2, at the beginning of the region of exponential amplification, to obtain Cq (quantification cycle) and the data obtained were exported into a MS Excel workbook (Microsoft Inc.) for further analysis.

### Statistical analysis

For the relation between the expressions of the different marker genes with the different best combination genes the relative quantity values were transformed into log_2_ (thus rendering them parametric) and tested through ANOVA in the program SAS 9 for Windows, SAS Institute Inc., Cary, NC, USA. When the p value of the ANOVA was lower than 0.05 a Tukey test was performed and statistically significant differences were accepted for a p value lower than 0.05.

## Competing interests

All authors declare they have no financial or non-financial competing interests.

## Authors’ contributions

JLC selection of the criteria to retrieve the reference genes from the microarray; statistical analysis; experimental greenhouse and lab work; MR statistical analysis of the Grapegen Affymetrix microarray; LC Application of the softwares and ΔCq method; writing the manuscript; SA project PI; revision of the manuscript. All authors read and approved the final manuscript.
